# Diabetes and hypertension markedly increased the risk of ischemic stroke associated with high serum resistin concentration in a general Japanese population: the Hisayama Study

**DOI:** 10.1186/1475-2840-8-60

**Published:** 2009-11-18

**Authors:** Haruhiko Osawa, Yasufumi Doi, Hideichi Makino, Toshiharu Ninomiya, Koji Yonemoto, Ryoichi Kawamura, Jun Hata, Yumihiro Tanizaki, Mitsuo Iida, Yutaka Kiyohara

**Affiliations:** 1 Department of Molecular and Genetic Medicine, Ehime University Graduate School of Medicine, Ehime, Japan; 2 Department of Medicine and Clinical Science, Graduate School of Medical Sciences, Kyushu University, Fukuoka, Japan; 3 Department of Environmental Medicine, Graduate School of Medical Sciences, Kyushu University, Fukuoka, Japan

## Abstract

**Background:**

Resistin, secreted from adipocytes, causes insulin resistance in mice. The relationship between resistin and coronary artery disease is highly controversial, and the information regarding resistin and ischemic stroke is limited. In the present study, the association between serum resistin concentration and cardiovascular disease (CVD) was investigated in a general Japanese population.

**Methods:**

A total of 3,201 community-dwelling individuals aged 40 years or older (1,382 men and 1,819 women) were divided into quintiles of serum resistin, and the association between resistin and CVD was examined cross-sectionally. The combined effect of either diabetes or hypertension and high serum resistin was also assessed. Serum resistin was measured using ELISA.

**Results:**

Compared to those without CVD, age- and sex-adjusted mean serum resistin concentrations were greater in subjects with CVD (p = 0.002) or ischemic stroke (p < 0.001), especially in those with lacunar and atherothrombotic infarction, but not elevated in subjects with hemorrhagic stroke or coronary heart disease. When analyzed by quintile of serum resistin concentration, the age- and sex-adjusted odds ratio (OR) for having CVD and ischemic stroke increased with quintile of serum resistin (p for trends, 0.02 for CVD, < 0.001 for ischemic stroke), while such associations were not observed for hemorrhagic stroke or coronary heart disease. Compared to the first quintile, the age- and sex-adjusted OR of ischemic stroke was greater in the third (OR = 3.54; 95% confidence interval [CI], 1.17-10.67; p = 0.02), fourth (OR = 4.48; 95% CI, 1.53-13.09; p = 0.006), and fifth quintiles (OR = 4.70; 95% CI, 1.62-13.61; p = 0.004). These associations remained substantially unchanged even after adjustment for other confounding factors including high-sensitivity C-reactive protein. In the stratified analysis, the combination of high serum resistin and either diabetes or hypertension markedly increased the risk of ischemic stroke.

**Conclusion:**

Elevated serum resistin concentration appears to be an independent risk factor for ischemic stroke, especially lacunar and atherothrombotic infarction in the general Japanese population. The combination of high resistin and the presence of either diabetes or hypertension increased the risk of ischemic stroke.

## Background

Resistin belongs to a family of cysteine-rich secretory proteins called resistin-like molecules [1]. In rodents, resistin is derived almost exclusively from adipose tissue, and serum resistin is elevated in animal models of obesity and insulin resistance [2-4]. On the other hand, in humans, monocytes and macrophages express resistin at high levels [5]; thus, the pathophysiological role of resistin may differ between species. *In vitro*, resistin activates human endothelial cells, leading to increased expression of adhesion molecules, and induces human aortic muscle cell proliferation [6,7]. Furthermore, several clinical and epidemiological studies have revealed positive associations between plasma concentrations of resistin and proinflammatory cytokines [8-10], which are emerging risk factors for cardiovascular disease (CVD). These findings suggest that resistin contributes to the development of atherosclerosis and thereby is linked to clinical vascular events. However, the relationship between resistin and coronary artery disease is highly controversial [11-14]. Furthermore, the information regarding resistin and ischemic stroke in general population is limited to only one epidemiological study that reported no association between circulating resistin and ischemic stroke [14].

The aim of the present study was to examine the association between serum resistin and CVD in a cross-sectional study of a defined Japanese population, taking into account a comprehensive list of risk factors, including high-sensitivity C-reactive protein (hs-CRP). Our findings suggest that elevated serum resistin concentration is a significant risk factor for ischemic stroke in a general Japanese population.

## Methods

### Study population

In 2002, a screening survey for the present study was performed in the town of Hisayama, a suburb of the Fukuoka metropolitan area in Japan. The age and occupational distributions and nutrient intake of the population were almost identical to those of Japan as a whole based on data from the national census and nutrition survey. A detailed description of this survey was published previously [15]. Briefly, of all residents aged 40 years or over, 3,328 underwent the examination (participation rate, 77.6%). After excluding 30 subjects who did not consent to participate in the study, 86 who had already eaten breakfast, and 11 who did not have enough stored sera with which to measure resistin concentrations, a total of 3,201 (1,382 men and 1,819 women) were enrolled in the study group and underwent a comprehensive assessment. This study was approved by the Ethics Committee of Kyushu University, and written informed consent was obtained from all participants.

### Definition of cardiovascular disease

Cases of CVD were defined as subjects who had histories of stroke or coronary heart disease. CVD was identified using the following criteria. The diagnosis and classification of stroke were determined on the basis of clinical information, including brain computed tomography and magnetic resonance imaging, cerebral angiography, echocardiography or carotid duplex imaging. Stroke was defined as sudden onset of nonconvulsive and focal neurological deficit persisting for ≥24 hours and was classified as either ischemic or hemorrhagic [16,17]. Hemorrhagic stroke included brain hemorrhage and subarachnoid hemorrhage. Ischemic stroke was further divided into 4 clinical categories -- lacunar infarction, atherothrombotic infarction, cardioembolic infarction, and undetermined subtype of ischemic stroke -- based on the Classification of Cerebrovascular Disease III proposed by the National Institute of Neurological Disorders and Stroke [18], as well as on the basis of the diagnostic criteria of the Trial of Org10172 in Acute Stroke Treatment Study [19] and the Cerebral Embolism Task Force [20]. Details of the diagnostic criteria for the ischemic stroke subtypes have been described previously [21]. In brief, lacunar infarction was defined as the presence of a relevant brainstem, basal ganglia, or subcortical hemispheric lesion with a diameter of < 1.5 cm demonstrated by brain imaging, and no evidence of cerebral cortical or cerebellar impairment. Atherothrombotic infarction was diagnosed when the subjects had significant stenosis (> 50%) or occlusion of a major cerebral artery with an infarct ≥1.5 cm in a brain imaging study. The diagnosis of cardioembolic infarction was made on the basis of primary and secondary clinical features suggestive of cardioembolic infarction as reported by the Cerebral Embolism Task Force [20]. The undetermined subtype of ischemic stroke included strokes that could not be classified because of insufficient clinical or morphologic information. We considered morphologic findings to be significant and used clinical features as reference information. Cases of cerebrovascular diseases that could be attributed to a distinct pathology, such as collagen disease, hematological disorder, trauma, chronic subdural hematoma, or moyamoya disease, were excluded from the evaluation.

The diagnostic criteria for coronary heart disease included acute myocardial infarction, silent myocardial infarction or coronary artery disease followed by coronary artery bypass surgery or angioplasty [16,17]. Acute myocardial infarction was diagnosed when a subject met at least two of the following criteria: (1) typical symptoms, including prolonged severe anterior chest pain; (2) abnormal elevation in cardiac enzymes, i.e., greater than a two-fold increase in the upper limit of the normal range; (3) evolving diagnostic electrocardiogram (ECG) changes; and, (4) morphological changes, including local asynergy of cardiac wall motion on echocardiography or persistent perfusion defects observed using cardiac scintigraphy. Silent myocardial infarction was defined as the above-mentioned morphological changes without any history of clinical symptoms or abnormalities in cardiac enzymes. Among the participants, 175 subjects had a history of CVD as follows: 79 had ischemic stroke; 41 had hemorrhagic stroke; 42 had coronary heart disease; 11 had both ischemic stroke and coronary heart disease; and, 2 had hemorrhagic stroke and coronary heart disease. In the analysis stratified by type of CVD, coexisting CVDs were each stratified into their respective types. Furthermore, on the basis of the above criteria, 90 ischemic stroke cases were divided into 51 cases of lacunar infarction, 29 of atherothrombotic infarction, 7 of cardioembolic infarction, and 3 of undetermined infarction; 43 hemorrhagic stroke cases were divided into 31 of brain hemorrhage and 12 of subarachnoid hemorrhage.

### Measurement of serum resistin concentrations

At the screening examination, blood samples were obtained between 8:00 and 10:30 AM after at least a 12-hour overnight fast. An aliquot of serum from each subject was stored at -80°C. Serum resistin was measured using a human ELISA kit (R&D Systems, Inc., Minneapolis, MN) following the manufacturer's protocol. According to data supplied by the manufacturer, the limit of detection was 0.16 ng/ml, and the intra-assay coefficient of variations (CVs) were < 5% for low concentrations, and < 4% for high concentrations. The inter-assay CV was < 9% for both low and high concentrations. Recovery was greater than 90%. We confirmed that linearity was maintained at concentrations less than 0.16 ng/ml, and both intra- and inter-assay CVs were comparable to those specified by the manufacturer (2.6-10.5%). The antibodies used in the enzyme-linked immunosorbent assay (ELISA) do not cross-react with either mouse resistin or other human cytokines. The results obtained using the ELISA manufactured by R&D were highly correlated with those obtained previously using a kit supplied by LINCO (Linco Research, Inc., St. Charles, MO) (r = 0.987, y = 1.040x+0.469, y, this kit, x, LINCO's kit) [22-24].

### Measurement of confounding factors

Blood for the glucose assay was collected into tubes containing NaF, and plasma glucose concentrations were determined immediately after venipuncture using the glucose-oxidase method. Serum total cholesterol and high density lipoprotein (HDL) cholesterol concentrations were determined enzymatically. hs-CRP concentrations were measured using a modification of the Behring Latex-Enhanced CRP assay on a Behring Nephelometer BN-100 (Behring Diagnostics, Westwood, MA). Blood pressure was measured three times using a standard mercury sphygmomanometer in the seated position after the subject had rested for at least 5 minutes. ECG abnormalities were defined as left ventricular hypertrophy (Minnesota Code 3-1) and/or ST depression (Minnesota Code 4-1, 2, 3).

Body height and weight were measured in light clothing without shoes, and body mass index (BMI) was calculated. Each participant completed a self-administered questionnaire that assessed medical history, smoking habits, alcohol intake, and exercise. The questionnaire was checked by trained interviewers at the screening. Alcohol intake and smoking habits were classified as either presence or absence of current habitual use. Those subjects who engaged in sports or other forms of exercise ≥3 times a week during their leisure time categorized regular exercisers.

### Statistical analysis

The SAS software package version 8.2 (SAS Institute, Inc., Cary, NC) was used to perform all statistical analyses. The subjects were divided into quintiles of resistin concentration as follows: < 6.8, 6.8 to 8.7, 8.8 to 11.5, 11.6 to 16.2, and ≥16.3 ng/ml. Values of possible risk factors were adjusted for age and sex using the covariance method and were compared among the quintiles using the linear regression model. Age- and sex-adjusted resistin concentrations were compared among CVD subtypes using the same methods. The frequencies of risk factors were adjusted for age and sex by the direct method using all subjects as a standard population and were tested for trends using the Cochran-Mantel-Haenszel test. Age- and sex-adjusted or multivariate-adjusted odds ratios (ORs) and 95% confidence intervals (CIs) for CVD were calculated using logistic regression analysis. A p-value < 0.05 was considered statistically significant in all analyses.

## Results

### Clinical characteristics of subjects

Age- and sex-adjusted means or frequencies of potential risk factors by quintiles of serum resistin concentration are shown in Table 1. Age and hs-CRP, and the frequency of male sex increased with quintiles of resistin, while mean HDL-cholesterol and the frequencies of alcohol consumption and regular exercise were negatively associated with the quintiles of resistin. The other variables were not significantly associated with resistin quintiles.

**Table 1:** Age- and sex-adjusted means or frequencies of cardiovascular risk factors according to serum resistin quintiles.

	Serum resistin level (ng/ml)					
	
	1.5-6.7	6.8-8.7	8.8-11.5	11.6-16.2	16.3-90.2	
Variables	(n = 648)	(n = 633)	(n = 641)	(n = 636)	(n = 643)	P value for trend
Age (years)	59 (11)	61 (12)	61 (12)	62 (13)	64 (14)	< 0.001
Men (%)	39.2	38.7	40.6	46.2	51.2	< 0.001
BMI (kg/m^2^)	23.1 (3.4)	23.2 (3.4)	22.9 (3.4)	23.1 (3.4)	23.1 (3.4)	0.69
Fasting plasma glucose (mmol/l)	6.1 (1.3)	6.1 (1.3)	6.0 (1.3)	6.1 (1.3)	6.0 (1.3)	0.55
Diabetes (%)	13.7	13.8	11.2	14.8	14.3	0.82
Total cholesterol (mmol/l)	5.25 (0.89)	5.22 (0.89)	5.27 (0.89)	5.27 (0.89)	5.19 (0.90)	0.51
HDL-cholesterol (mmol/l)	1.70 (0.40)	1.61 (0.39)	1.60 (0.39)	1.60 (0.39)	1.53 (0.40)	< 0.001
High sensitivity C-reactive protein (mg/l)	0.43 (0.04-4.53)	0.49 (0.05-5.06)	0.54 (0.05-5.53)	0.55 (0.05-5.64)	0.68 (0.07-7.18)	< 0.001
Systolic blood pressure (mmHg)	132 (20)	133 (20)	131 (20)	132 (20)	132 (20)	0.65
Diastolic blood pressure (mmHg)	79 (12)	79 (12)	78 (12)	79 (12)	78 (12)	0.38
Antihypertensive medication (%)	21.7	28.2	22.1	22.8	26.2	0.52
Hypertension (%)	44.4	46.6	39.6	43.6	47.2	0.67
Electrocardiographic abnormalities (%)	15.1	16.1	15.8	14.9	16.1	0.47
Current drinking (%)	50.9	45.1	40.8	42.1	37.1	< 0.001
Current smoking (%)	20.9	20.2	22.7	20.3	24.7	0.14
Regular exercise (%)	11.6	11.0	10.8	8.4	9.5	0.04

BMI: body mass index; HDL: high density lipoprotein. All values are given as mean (standard deviation) or as percentages.

C-reactive protein is shown by geometric mean and 95% confidence intervals due to the skewed distribution. Age and percent of men are not adjusted.

### Serum resistin concentrations by cardiovascular disease

Age- and sex-adjusted serum resistin concentrations were greater in subjects with total CVD than in those without CVD (p = 0.002) (Table 2). When CVD was divided into types, subjects with ischemic stroke had greater resistin concentrations than those without CVD (p < 0.001). Regarding subtypes of ischemic stroke, subjects with lacunar and atherothrombotic infarction had greater resistin than subjects without CVD (p = 0.02 for lacunar infarction; p < 0.001 for atherothrombotic infarction); however, no association was observed between resistin and cardioembolic infarction or undetermined subtype of ischemic stroke. Resistin concentrations in subjects with hemorrhagic stroke, including brain and subarachnoid hemorrhage, and coronary heart disease were not different from subjects without CVD.

**Table 2:** Age- and sex-adjusted resistin values according to types of cardiovascular disease.

	Number	Age- and sex- adjusted resistin values (ng/ml)	P vs. no cardiovascular disease cases
No cardiovascular disease	3,026	10.3 (3.8-28.0)	-
Cardiovascular disease	175	11.7 (4.2-32.4)	0.002
Ischemic stroke	90	13.0 (4.7-35.7)	< 0.001
Lacunar infarction	51	12.1 (4.4-32.9)	0.02
Atherothrombotic infarction	29	16.3 (6.0-44.4)	< 0.001
Cardioembolic infarction	7	11.5 (4.2-31.0)	0.55
Undetermined infarction	3	6.7 (1.7-18.0)	0.14
Hemorrhagic stroke	43	10.3 (3.8-28.1)	0.90
Brain hemorrhage	31	11.1 (4.1-30.2)	0.37
Subarachnoid hemorrhage	12	8.6 (3.2-23.2)	0.22
Coronary heart disease	55	11.3 (4.1-31.0)	0.14

Resistin is shown by geometric mean and 95% confidenceintervals due to the skewed distribution.

### Association of resistin concentrations with cardiovascular disease

**Figure 1:**
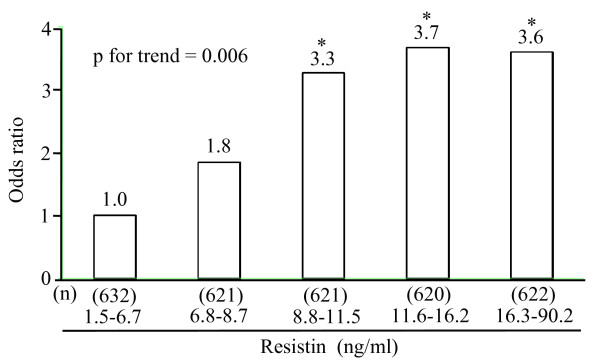
**Multivariate-adjusted odds ratio for the presence of ischemic stroke according to quintiles of serum resistin concentrations**. The OR compared to the first quintile is shown. Multivariate adjustment was made for age, sex, body mass index, diabetes, total cholesterol, high density lipoprotein cholesterol, high sensitivity C-reactive protein, systolic blood pressure, electrocardiographic abnormalities, current drinking, current smoking and regular exercise. *, p < 0.05 compared to the first quartile.

To further evaluate the association between serum resistin concentrations and the risk of CVD, age- and sex-adjusted or multivariate-adjusted ORs were estimated by quintiles of resistin concentration (Table 3). In Model 1, the age- and sex-adjusted ORs for total CVD and ischemic stroke increased with quintile of serum resistin (p for trends, 0.02 for CVD, < 0.001 for ischemic stroke). Compared to the first quintile, the age- and sex-adjusted OR for ischemic stroke was greater in the third, fourth, and fifth quintiles (the third quintile: OR 3.54, 95%CI 1.17-10.67, p = 0.02; the fourth quintile: OR 4.48, 95%CI 1.53-13.09, p = 0.006; the fifth quintile: OR 4.70, 95%CI 1.62-13.61, p = 0.004), while there was no association between quintile of resistin and CVD. The association between quintile of resistin and ischemic stroke remained significant even after adjustment for age, sex, BMI, diabetes, total cholesterol, HDL-cholesterol, systolic blood pressure, ECG abnormalities, current drinking, current smoking, and regular exercise (Model 2). Furthermore, as shown in Model 3, after adjustment for hs-CRP and the confounding factors, this association remained essentially unchanged (Fig. 1). No significant associations were found between quintile of resistin and either hemorrhagic stroke or coronary heart disease.

**Table 3:** Age- and sex- or multivariate-adjusted odds ratios for cardiovascular diseases according to serum resistin quintiles.

		Serum resistin level (ng/ml)					
	
		1.5-6.7	6.8-8.7	8.8-11.5	11.6-16.2	16.3-90.2	P value for trend
No cardiovascular disease, n		628	610	604	595	589	
Cardiovascular disease, n		20	23	37	41	54	
Model 1-adjusted OR (95% CI)		1 (referent)	0.95 (0.51-1.76)	1.55 (0.88-2.74)	1.51 (0.86-2.64)	1.64 (0.95-2.84)	0.02
Model 2-adjusted OR (95% CI)		1 (referent)	0.88 (0.47-1.66)	1.56 (0.88-2.77)	1.39 (0.79-2.45)	1.52 (0.87-2.66)	0.04
Model 3-adjusted OR (95% CI)		1 (referent)	0.87 (0.46-1.63)	1.53 (0.86-2.72)	1.36 (0.77-2.41)	1.47 (0.84-2.58)	0.06
Ischemic stroke, n		4	11	17	25	33	
Model 1-adjusted OR (95% CI)		1 (referent)	2.21 (0.70-7.05)	3.54 (1.17-10.67)	4.48 (1.53-13.09)	4.70 (1.62-13.61)	< 0.001
Model 2-adjusted OR (95% CI)		1 (referent)	1.94 (0.60-6.23)	3.36 (1.11-10.22)	3.87 (1.31-11.39)	3.97 (1.36-11.61)	0.002
Model 3-adjusted OR (95% CI)		1 (referent)	1.84 (0.57-5.96)	3.26 (1.07-9.92)	3.66 (1.24-10.79)	3.59 (1.22-10.55)	0.006
Hemorrhagic stroke, n		9	7	9	7	11	
Model 1-adjusted OR (95% CI)		1 (referent)	0.70 (0.26-1.92)	0.93 (0.36-2.37)	0.66 (0.24-1.80)	0.89 (0.36-2.23)	0.83
Model 2-adjusted OR (95% CI)		1 (referent)	0.70 (0.26-1.92)	1.02 (0.39-2.64)	0.70 (0.25-1.93)	0.98 (0.38-2.50)	0.99
Model 3-adjusted OR (95% CI)		1 (referent)	0.69 (0.25-1.90)	1.00 (0.39-2.60)	0.69 (0.25-1.90)	0.96 (0.37-2.46)	0.97
Coronary heart disease, n		9	6	12	11	17	
Model 1-adjusted OR (95% CI)		1 (referent)	0.55 (0.19-1.57)	1.13 (0.47-2.73)	0.88 (0.36-2.18)	1.11 (0.48-2.59)	0.48
Model 2-adjusted OR (95% CI)		1 (referent)	0.48 (0.16-1.41)	1.20 (0.49-2.95)	0.79 (0.31-1.99)	1.05 (0.44-2.50)	0.53
Model 3-adjusted OR (95% CI)		1 (referent)	0.49 (0.17-1.42)	1.21 (0.49-2.97)	0.80 (0.32-2.02)	1.09 (0.45-2.59)	0.49

OR: odds ratio; CI: confidence interval. Model 1: adjustment was made for age and sex; Model 2: adjustment was made for age, sex, body mass index, diabetes, total cholesterol, HDL cholesterol, systolic blood pressure, electrocardiographic abnormalities, current drinking, current smoking, and regular exercise; Model 3: adjustment was made for the variables used in Model 2 and for high sensitivity C-reactive protein.

### Combined effects of high serum resistin with other risk factors

**Figure 2:**
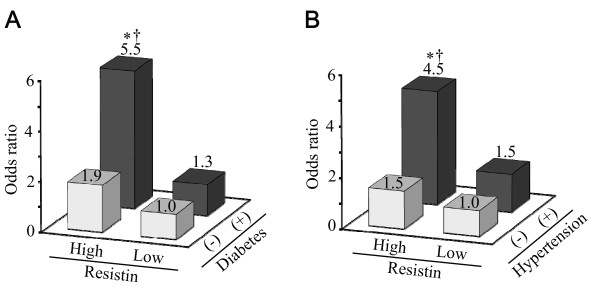
**Multivariate-adjusted odds ratios for ischemic stroke according to the presence or absence of high serum resistin concentrations and either diabetes or hypertension**. Multivariate adjustment was made as described in the legend of Fig. 1, but each risk factor that had been used for categorization was excluded from the confounding factors. High resistin was defined as the third, fourth, and fifth quintile of resistin concentration. A. Diabetes (DM). The OR compared to the reference (DM (-) + low resistin) is shown. *, OR = 5.54 (95% CI, 2.60-11.76), p < 0.001 for DM (+) + high resistin. OR = 1.87 (95% CI, 0.97-3.59), p = 0.06, for DM (-) + high resistin; OR = 1.25 (95% CI, 0.34-4.64), p = 0.73 for DM (+) + low resistin. †, p < 0.05 compared to DM (+) + low resistin. B. Hypertension (HT). OR compared to the reference (HT (-) + low resistin) is shown. *, OR=4.46 (95% CI, 1.71-11.62), p = 0.002 for HT (+) + high resistin. OR= 1.47 (95% CI, 0.53-4.08), p = 0.45, for HT (-) + high resistin; OR = 1.48 (95% CI, 0.49-4.53), p = 0.49 for HT (+) + low resistin. †, p < 0.05 compared to HT (+) + low resistin.

To determine whether high serum resistin concentration was associated with increased risk of ischemic stroke in subjects who had either diabetes or hypertension, we examined the combined and unique effects of high resistin and the presence of either diabetes or hypertension on the risk of ischemic stroke. High resistin was defined as the third, fourth, and fifth quintile of resistin concentration. Compared to non-diabetic subjects with low resistin, the age- and sex-adjusted OR of ischemic stroke was greater in non-diabetic subjects with high resistin (OR: 2.15; 95% CI: 1.13-4.10; p = 0.02), but not in diabetic subjects with low resistin (OR: 1.27; 95% CI: 0.35-4.62; p = 0.72). The risk of ischemic stroke markedly increased in diabetic subjects with high resistin (OR: 5.88; 95% CI: 2.82-12.26; p < 0.001). Furthermore, diabetic subjects with high resistin had greater risk of ischemic stroke than diabetic subjects with low resistin (p < 0.05). Similarly, a marked increase in the age- and sex-adjusted risk of ischemic stroke was observed in hypertensive subjects with high resistin compared with normotensive subjects with low resistin (OR: 4.66; 95% CI: 1.81-11.97; p = 0.001), but not in normotensive subjects with high resistin (OR: 1.87; 95%CI: 0.69-5.10; p = 0.22), or hypertensive subjects with low resistin (OR: 1.52; 95%CI: 0.51-4.54; p = 0.45). In hypertensive subjects, high resistin was associated with risk of ischemic stroke (p < 0.05). These associations remained robust even after adjustment for the above-mentioned confounding factors (Fig. 2).

## Discussion

Our large cross-sectional data set representative of the general Japanese population demonstrated that serum resistin concentrations were greater in subjects with ischemic stroke, especially in those with lacunar and atherothrombotic infarction. Elevated resistin was an independent risk factor for ischemic stroke even after adjustment for confounding factors including hs-CRP. In the stratified analyses, the combination of high resistin and either diabetes or hypertension markedly increased the risk of ischemic stroke.

In our study, subjects with lacunar and atherothrombotic infarction, but not cardioembolic infarction, had greater resistin concentrations. In particular, in subjects who had suffered atherothrombotic infarction, serum resistin was dramatically elevated. Atherothrombotic infarction is caused by atherosclerotic lesions of large vessels, while lacunar infarcts occur as a result of multiple mechanisms: 1) lipohyalinosis and/or fibrinoid necrosis; 2) microatheroma; 3) atherosclerosis of the basilar and middle cerebral artery stem or proximal division of large vessels; or, 4) cardioembolic occlusion [25]. Lipohyalinosis is a vasculopathy caused by hypertension [25]. In contrast, large vessel atherosclerosis is associated with traditional risk factors and inflammation, while cardioembolism seems less related to atherosclerosis. Thus, our findings regarding the association between each subtype of ischemic stroke and serum resistin is reasonably consistent with the known pathology. Most recently, Tsukahara et al. reported that serum resistin levels in individuals with diabetes who had a history of stroke were higher than those without in a case/control study of Japanese [26]. However, a recent nested case-control epidemiological study of Europeans showed that elevated resistin concentration was not a predictor of ischemic stroke [14]. This inconsistency might be caused by differences in study design and ethnicity. Although the European study lacked information on ischemic stroke subtype, in general, the prevalence of cardioembolic infarction, which is a potentially fatal disease and weakly associated with atherosclerosis in brain vessels, may be greater in prospective design than in cross-sectional design. In addition, Caucasians are at a greater risk of cardioembolic infarction compared with Japanese individuals [16,27,28].

The present study showed that the association between circulating resistin and ischemic stroke was independent of hs-CRP and other confounding factors. Resistin could directly stimulate expression of pro-inflammatory cytokines such as tumor necrosis factor-α, interleukin-6 in human peripheral blood mononuclear cells [29] and increase vascular cell adhesion molecule-1 and intracellular adhesion molecule-1 gene expression in vascular endothelial cells [7]. A recent clinical study showed that the association between plasma resistin and monocyte chemoattractant protein-1 was independent of hs-CRP and other confounding factors [30]. Therefore, resistin could potentiate vascular inflammation and atherogenesis.

Our previous studies demonstrated that serum resistin was tightly correlated with a single nucleotide polymorphism (SNP) at -420 of the human resistin gene, namely, resistin was highest in individuals with the G/G genotype, followed by the C/G and C/C genotypes [22-24]. It was reported that Japanese individuals with the C/G or G/G genotypes of SNP-420 were more likely to have had a stroke than those with the C/C genotype [26]. A clinical study of Finns found that the G/G genotype was associated with cerebrovascular disease [31]. Thus, resistin may directly contribute to ischemic stroke.

In the present study, serum resistin was not associated with hemorrhagic stroke or coronary heart disease. To our knowledge, there are no published studies that examined the relationship between resistin and hemorrhagic stroke. Regarding coronary heart disease, reported findings have been highly controversial; some epidemiological and clinical studies showed a significant association between elevated resistin and coronary heart disease [13,14], whereas, in other studies, this association was not observed [11,12]. Further investigations, especially those involving a larger number of samples, are necessary to clarify the role of resistin in the development of hemorrhagic stroke and coronary artery disease.

The present study has three limitations that should be mentioned. First, due to the cross-sectional design, we cannot exclude the possibility that hyperresistinemia was a consequence of ischemic stroke. Prospective studies are required to address this critical question. Second, the number of subjects with ischemic stroke was relatively small, although we used a highly accurate method of detecting and classifying all cases. Likewise, the lack of association between either hemorrhagic stroke or coronary heart disease and serum resistin may be reflection of the small samples. Third, since hemorrhagic stroke and coronary heart disease both have high mortality, survival bias could partly explain why no association was found between these diseases and serum resistin. Thus, the generalizability of the study results may be somewhat limited.

## Conclusion

Serum resistin concentration was a risk factor for ischemic stroke, especially lacunar and atherothrombotic infarction, and the association was independent of traditional confounding factors and hs-CRP. Furthermore, the combination of high resistin concentration and the presence of either diabetes or hypertension markedly increased the risk of ischemic stroke. The mechanism by which resistin induces ischemic stroke in humans and how resistin interacts with diabetes or hypertension to further increase the risk of ischemic stroke remain unclear. Further studies are required to clarify these issues.

## Competing interests

The authors declare that they have no competing interests.

## Authors' contributions

HO, YD, HM, and YK designed the study and directed its implementation, including quality assurance and control. TN and KY designed the study's analytic strategy. MI helped supervise the field activities. RK, JH, and YT helped conduct the literature review. All authors read and approved the final manuscript.
